# Supplementary material for: The Potential of Plum Seed Residue: Unraveling the Effect of Processing on Phytochemical Composition and Bioactive Properties
Source: Int J Mol Sci. 2024 Jan 19;25(2):0. doi: 10.3390/ijms25021236 (PMC11154296; doi:10.3390/ijms25021236)
Supplement: Supplementary file 1 [file ijms-25-01236-s001.zip › ijms-2810225-supplementary.pdf]

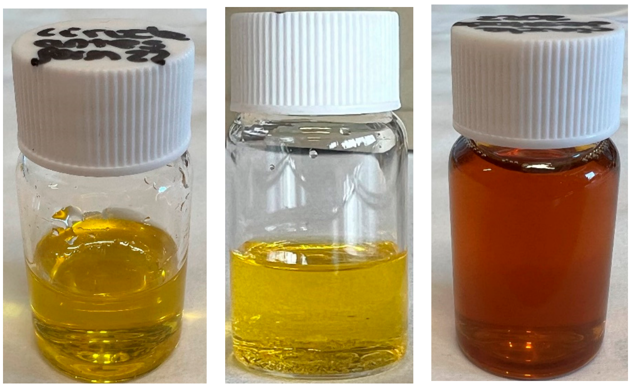

(**a**) (**b**) (**c**)

**Figure S1.** Pictures of plum seed oils obtained in the different stages of plum brandy processing. **a**) plum seed oil before fermentation, **b)** plum seed oil after fermentation, **c)** plum seed oil after both fermentation and distillation.

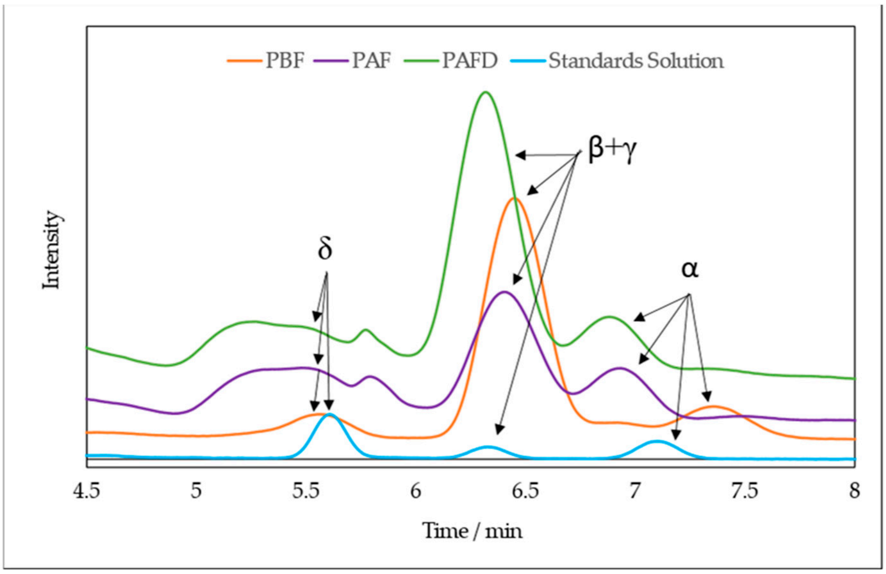


**Figure S2.** Comparative chromatogram at 292 nm between tocopherols in plum seed oils and standards solution by liquid chromatography coupled to ultraviolet-visible spectroscopy (HPLC-PDA). Intensity scale has been shifted for all signals. PAFD: plum seed oil after both fermentation and distillation; PAF: plum seed oil after fermentation; PBF: plum seed oil before fermentation. Standards solution at 5.0, 2.0 and 10.0 mg·L$^{-1}$ of α-, γ- and δ-tocopherol, respectively. Peaks were identified by spiking standards solution onto the sample.

**Table S1.** Identification of phenolic compounds by high-resolution liquid chromatography coupled to a quadrupole time-of-flight mass spectrometer (HPLC-ESI-QTOF-MS) in plum seed phenolic extracts.

| Peak Number | Compound | RT (min) | Molecular Formula | [M-H]$^-$ (m/z) | MS/MS Fragments (m/z) |
|---|---|---|---|---|---|
| 1 | Gallic acid [a] | 2.7 | $C_7H_6O_5$ | 169.1128 | **125.0306**; 97.0302; 79.0243; 51.0305 |
| 2 | 2,3-Dihydroxybenzoic acid [a] | 4.9 | $C_7H_6O_4$ | 153.1138 | 125.5103; **109.0303**; 91.0201; 65.006 |
| 3 | Neochlorogenic acid [b] | 4.9 | $C_{16}H_{18}O_9$ | 353.0879 | **191.0433**; 179.0335; 161.1005; 135.0433; 107.0429 |
| 4 | Protocatechuate[c] | 5.0 | $C_7H_6O_4$ | 153.0194 | 153.0159;120.5030; 109.0351; 91.0221; 81.0410 |
| 5 | Catechin[a] | 7.5 | $C_{15}H_{14}O_6$ | 289.2609 | 289.0584; 245.0703; 203.0567; 151.0285; 109.0185; 57.0262 |

| | | | | | |
|---|---|---|---|---|---|
| 6 | Chlorogenic acid [a] | 7.8 | $C_{16}H_{18}O_9$ | 353.3015 | 319.6037; 257.5812, **191.0613**; 161.0299; 85.0372 |
| 7 | Vanillic acid [a] | 9.2 | $C_8H_8O_4$ | 167.0351 | 151.0007; **108.0227**; 91.0128; 80.5217 |
| 8 | Epicatechin[a] | 9.8 | $C_{15}H_{14}O_6$ | 289.2609 | 289.0701; 245.0839; 203.0711; 151.0428; 109.0343; 57.0379 |
| 9 | Caffeic acid [a] | 9.6 | $C_9H_8O_4$ | 179.1502 | 135.0469; 117.0299, 89.0406; 65.0374 |
| 10 | Syringic acid [a] | 9.7 | $C_9H_{10}O_5$ | 197.0456 | 181.0194; 167.0176; 151.9915;138.0294;**123.0072**; 95.0121 |
| 11 | *p*-Coumaric acid [a] | 13.8 | $C_9H_8O_3$ | 163.1508 | **119.0516**; 93.0364; 65.0359 |
| 12 | *trans*-Ferulic acid [a] | 15.2 | $C_{10}H_{10}O_4$ | 193.1768 | 150.0655; **134.0400**; 106.0488; 89.0389 |
| 13 | Kaempferol 3-rutinoside [b] | 17.7 | $C_{27}H_{30}O_{15}$ | 593.1513 | 423.0438; 367.7453; **285.0372**; 195.1208; 61.9900 |
| 14 | Isorhamnetin 3-rutinoside [c] | 17.9 | $C_{28}H_{32}O_{16}$ | 623.1618 | 577.2840; **315.0504**; 300.0258; 271.6939; 243.0098 |
| 15 | Hesperidin[a] | 18.4 | $C_{28}H_{34}O_{15}$ | 609.5534 | 609.1852; 527.2731; 459.0930; 301.0707; 257.0955; 136.0119 |
| 16 | Quercetin [a] | 27.0 | $C_{15}H_{10}O_7$ | 301.2285 | 273.0425; 229.0516; 179.0033; **151.0081**; 121.0317 |
| 17 | Kaempferol [a] | 32.6 | $C_{15}H_{10}O_6$ | 285.2291 | 255.0218; 229.0400; 185.0529; 151.0335; **93.0308** |

Bold fragments are the ones, employed for quantitation purposes. Compared with MS data obtained from standard [a], FooDB database [b], and MassBank database [c]. RT means retention time.

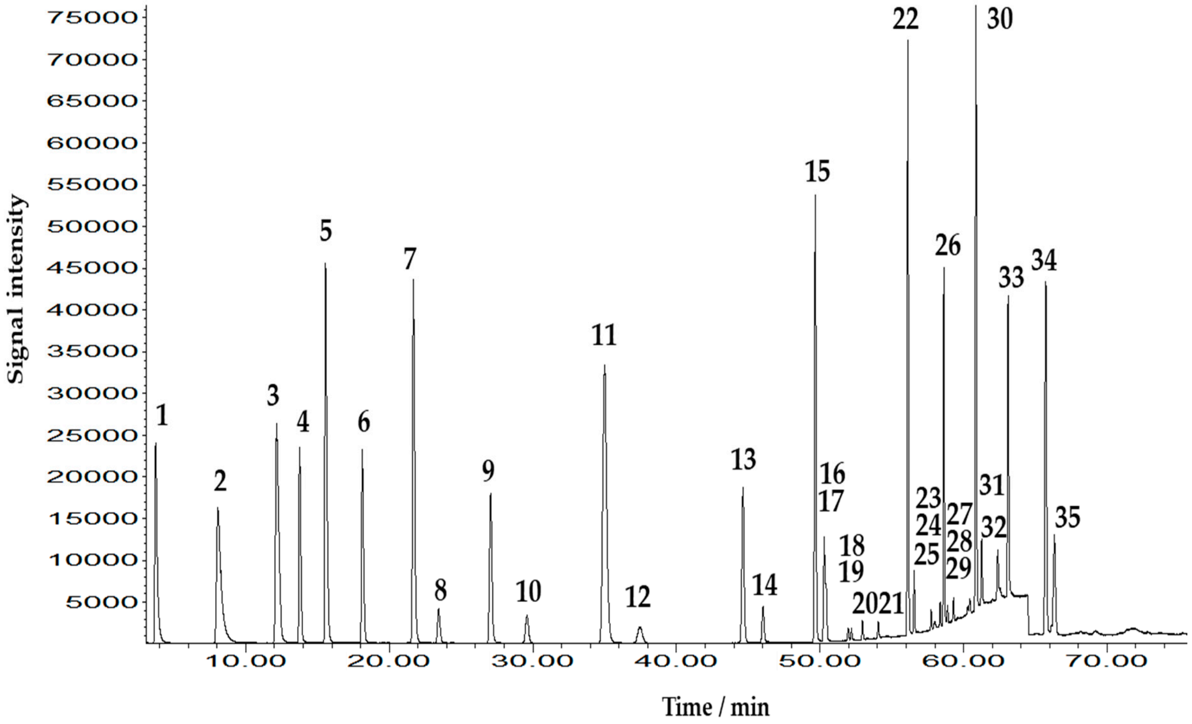


**Figure S3.** Chromatogram of the standard mixture of FAME 37 component SUPELCO Ref CRM47885 by gas chromatography coupled to mass spectrometry (GC- MS). Peak identification: (**1**) methyl hexanoate (C6:0; 400 μg·mL$^{-1}$); (**2**) methyl octanoate (C8:0; 400 μg·mL$^{-1}$); (**3**) methyl decanoate (C10:0; 400 μg·mL$^{-1}$); (**4**) methyl undecanoate (C11:0; 200 μg·mL$^{-1}$); (**5**) methyl laurate (C12:0; 400 μg·mL$^{-1}$); (**6**) methyl tridecanoate (C13:0; 200 μg·mL$^{-1}$); (**7**) methyl myristate (C14:0; 400 μg·mL$^{-1}$); (**8**) methyl myristoleate (C14:1n5; 200 μg·mL$^{-1}$); (**9**) methyl pentadecanoate (C15:0; 200 μg·mL$^{-1}$); (**10**) methyl cis-10-pentadecenoate (C15:1n5; 200 μg·mL$^{-1}$); (**11**) methyl palmitate (C16:0; 600 μg·mL$^{-1}$); (**12**) methyl palmitoleate (C16:1n7; 200 μg·mL$^{-1}$); (**13**) methyl heptadecanoate (margarate, C17:0; 200 μg·mL$^{-1}$); (**14**) cis-10-heptadecanoic acid methyl ester (C17:1n7c; 200 μg·mL$^{-1}$); (**15**) methyl stearate (C18:0; 400 μg·mL$^{-1}$); (**16**) cis-9-oleic acid methyl ester (C18:1n9c; 400 μg·mL$^{-1}$); (**17**) trans-9-elaidic

acid methyl ester (C18:1n9t; 200 µg·mL$^{-1}$); (**18**) methyl linolelaidate (C18:2n6t; 200 µg·mL$^{-1}$); (**19**) methyl linoleate (C18:2n6c; 200 µg·mL$^{-1}$); (**20**) methyl γ-linolenate (C18:3n6; 200 µg·mL$^{-1}$); (**21**) methyl linolenate (C18:3n3; 200 µg·mL$^{-1}$); (**22**) methyl arachidate (C20:0; 400 µg·mL$^{-1}$); (**23**) methyl cis-11-eicosenoate (C20:1n9; ≤ 200 µg·mL$^{-1}$); (**24**) cis 11,14-Eicosadienoic acid methyl ester (C20:2n6; 200 µg·mL$^{-1}$); (**25**) cis-8,11,14-eicosatrienoic acid methyl ester (C20:3n6; 200 µg·mL$^{-1}$); (**26**) methyl heneicosanoate (C21:0; 200 µg·mL$^{-1}$); (**27**) cis-5,8,11,14-eicosatetraenoic acid methyl ester (C20:4n6; 200 µg·mL$^{-1}$); (**28**) cis-11,14,17-eicosatrienoic acid methyl ester (C20:3n3; 200 µg·mL$^{-1}$); (**29**) cis-5,8,11,14,17-Eicosapentaenoic acid methyl ester (C20:5n3; 200 µg·mL$^{-1}$); (**30**) methyl behenate (C22:0; 400 µg·mL$^{-1}$); (**31**) methyl erucate (C22:1n9; 200 µg·mL$^{-1}$); (**32**) cis-13,16-docosadienoic acid methyl ester (C22:2n6; 200 µg·mL$^{-1}$); (**33**) methyl tricosanoate (C23:0; 200 µg·mL$^{-1}$); (**34**) methyl lignocerate (C24:0; 400 µg·mL$^{-1}$); (**35**) methyl nervonate (C24:1n9; 200 µg·mL$^{-1}$).

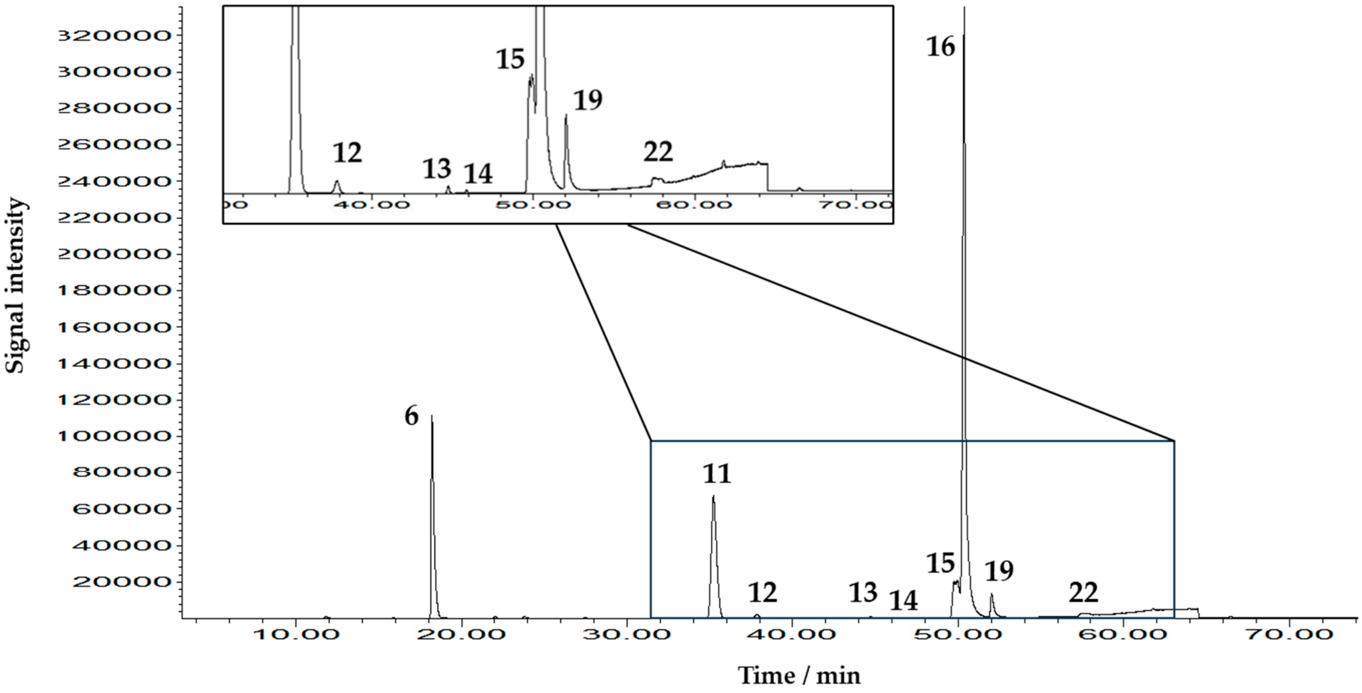


**Figure S4.** Chromatogram of plum seed oil before fermentation (PBF), which includes an ampliation aiming for easier appreciation of the peaks. Peak identification: (**6**) methyl methyl tridecanoate (C13:0) internal standard; (**11**) methyl palmitate (C16:0); (**12**) methyl palmitoleate (C16:1n7); (**13**) methyl heptadecanoate (margarate, C17:0); (**14**) cis-10-heptadecanoic acid methyl ester (C17:1n7c); (**15**) methyl stearate (C18:0); (**16**) cis-9-oleic acid methyl ester (C18:1n9c); (**19**) methyl linoleate (C18:2n6c); (**22**) methyl arachidate (C20:0).

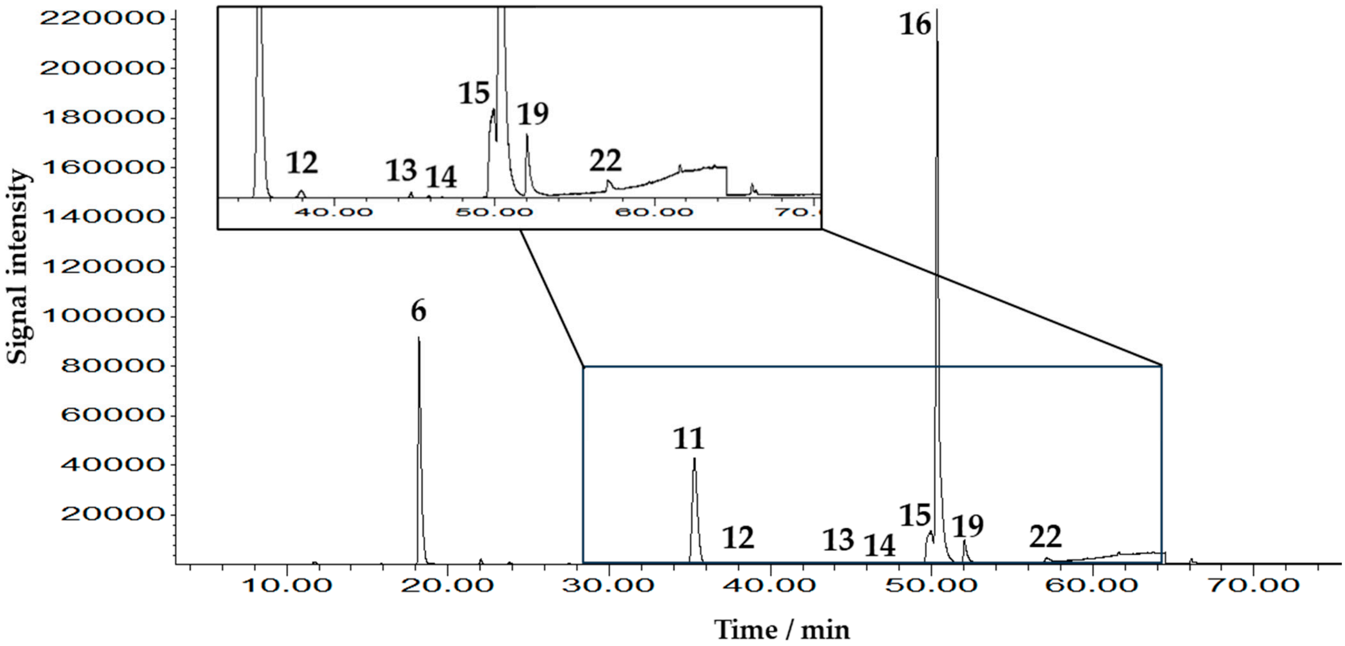


**Figure S5.** Chromatogram of plum seed oil after fermentation (PAF), which includes an ampliation aiming for easier appreciation of the peaks. Peak identification: (**6**) methyl methyl tridecanoate (C13:0) internal standard; (**11**) methyl palmitate (C16:0); (**12**) methyl palmitoleate (C16:1n7); (**13**) methyl heptadecanoate (margarate, C17:0); (**14**) cis-10-heptadecanoic acid methyl ester (C17:1n7c); (**15**) methyl stearate (C18:0); (**16**) cis-9-oleic acid methyl ester (C18:1n9c); (**19**) methyl linoleate (C18:2n6c); (**22**) methyl arachidate (C20:0).

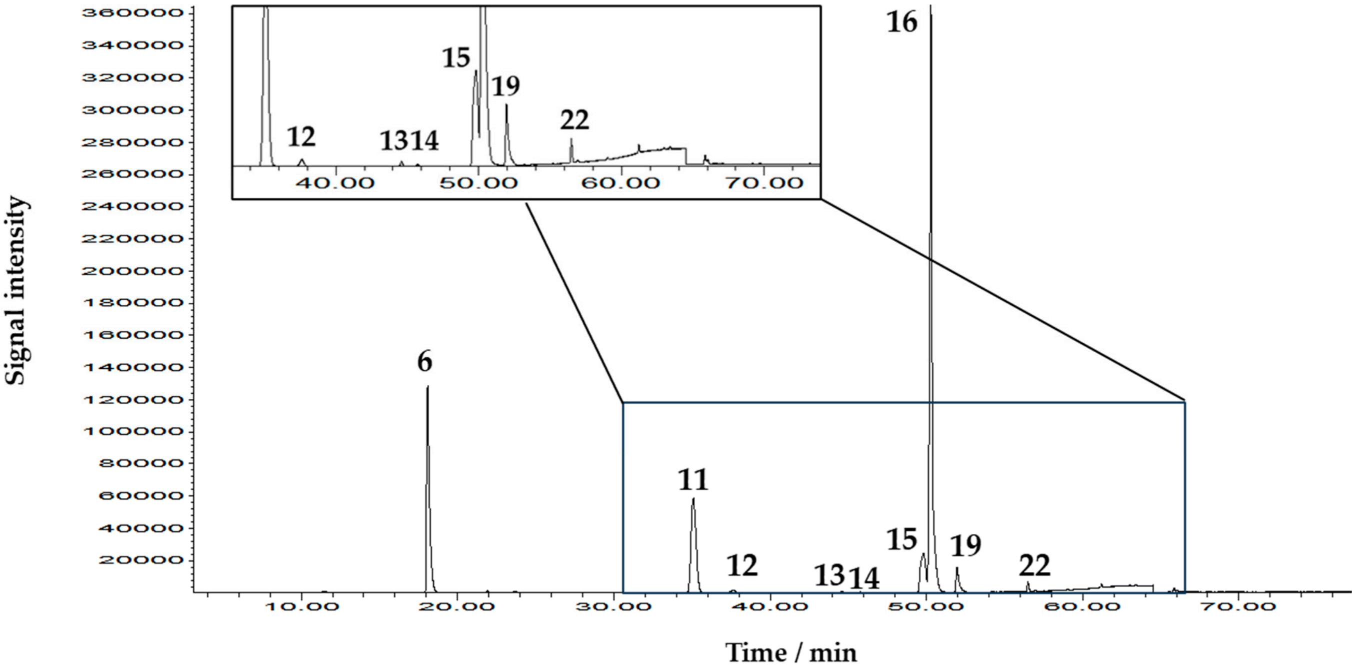


**Figure S6.** Chromatogram of plum seed oil after both fermentation and distillation (PAFD), which includes an ampliation aiming for easier appreciation of the peaks. Peak identification: (**6**) methyl methyl tridecanoate (C13:0) internal standard; (**11**) methyl palmitate (C16:0); (**12**) methyl palmitoleate (C16:1n7); (**13**) methyl heptadecanoate (margarate, C17:0); (**14**) cis-10-heptadecanoic acid methyl ester (C17:1n7c); (**15**) methyl stearate (C18:0); (**16**) cis-9-oleic acid methyl ester (C18:1n9c); (**19**) methyl linoleate (C18:2n6c); (**22**) methyl arachidate (C20:0).

**Table S2.** Response factors and limits of detection and quantification of FAME 37 standard mixture SUPELCO Ref CRM47885 by gas chromatography coupled to mass spectrometry (GC- MS).

| Compound | m/z relation | RF | RSD (%) | LOD ($\mu g \cdot g^{-1}$) | LOQ ($\mu g \cdot g^{-1}$) |
|---|---|---|---|---|---|
| Methyl hexanoate (C6:0) | 74 | 1.2 | 11.6 | 13.3 | 40.3 |
| Methyl octanoate (C8:0) | 74 | 1.4 | 11.0 | 24.5 | 74.3 |
| Methyl decanoate (C10:0) | 74 | 1.7 | 11.2 | 5.5 | 16.5 |
| Methyl undecanoate (C11:0) | 74 | 1.7 | 9.4 | 2.8 | 8.4 |
| Methyl laurate (C12:0) | 74 | 1.8 | 6.6 | 4.7 | 14.2 |
| **Methyl tridecanoate (C13:0)** | **74** | **1.8** | **5.3** | **2.9** | **8.8** |
| Methyl myristate (C14:0) | 74 | 1.75 | 2.26 | 4.0 | 12.1 |
| Methyl myristoleate (C14:1n5) | 74 | 0.38 | 4.57 | 27.3 | 82.8 |
| Methyl pentadecanoate (C15:0) | 74 | 1.65 | 3.01 | 3.4 | 10.4 |
| Methyl cis-10-pentadecenoate (C15:1n5) | 74 | 0.37 | 4.67 | 18.4 | 55.6 |
| **Methyl palmitate (C16:0)** | **74** | **1.7** | **3.8** | **7.3** | **22.1** |
| **Methyl palmitoleate (C16:1n7)** | **74** | **0.35** | **6.45** | **21.6** | **65.6** |
| **Methyl heptadecanoate (margarate, C17:0)** | **74** | **1.6** | **5.5** | **3.8** | **11.4** |
| **Cis-10-heptadecanoic acid methyl ester (C17:1n7c)** | **74** | **0.34** | **4.69** | **14.2** | **43.0** |
| **Methyl stearate (C18:0)** | **74** | **1.7** | **5.8** | **4.9** | **14.9** |
| **Cis-9-oleic acid methyl ester (C18:1n9c)** | **74** | **0.36** | **1.46** | **20.7** | **62.7** |
| Trans-9-elaidic acid methyl ester (C18:1n9t) | 74 | 0.34 | 7.44 | 16.1 | 48.7 |
| Methyl linolelaidate (C18:2n6t) | 74 | 0.071 | 5.186 | 31.4 | 95.2 |
| **Methyl linoleate (C18:2n6c)** | **74** | **0.07** | **7.41** | **29.6** | **89.7** |
| Methyl $\gamma$-linolenate (C18:3n6) | 74 | 0.067 | 5.220 | 61.3 | 185.6 |
| Methyl linolenate (C18:3n3) | 74 | 0.069 | 6.742 | 54.8 | 165.9 |
| **Methyl arachidate (C20:0)** | **74** | **1.7** | **7.6** | **10.4** | **31.6** |
| Methyl cis-11-eicosenoate (C20:1n9) | 74 | 0.34 | 6.75 | 41.5 | 125.8 |
| Cis 11,14-Eicosadienoic acid methyl ester (C20:2n6) | 74 | 0.10 | 7.70 | 71.5 | 216.8 |
| Cis-8,11,14-eicosatrienoic acid methyl ester (C20:3n6) | 74 | 0.064 | 3.813 | 54.5 | 165.2 |
| Methyl heneicosanoate (C21:0) | 74 | 1.7 | 8.3 | 7.8 | 23.6 |
| Cis-5,8,11,14-eicosatetraenoic acid methyl ester (C20:4n6) | 74 | 0.09 | 9.53 | 85.3 | 258.6 |
| Cis-11,14,17-eicosatrienoic acid methyl ester (C20:3n3) | 74 | 0.09 | 6.86 | 66.9 | 202.7 |
| Cis-5,8,11,14,17-Eicosapentaenoic acid methyl ester (C20:5n3) | 74 | 0.08 | 7.17 | 95.9 | 290.7 |
| Methyl behenate (C22:0) | 74 | 1.8 | 8.1 | 12.1 | 36.7 |
| Methyl erucate (C22:1n9) | 74 | 0.36 | 7.32 | 33.0 | 100.0 |
| Cis-13,16-docosadienoic acid methyl ester (C22:2n6) | 74 | 0.16 | 19.93 | 79.8 | 241.9 |
| Methyl tricosanoate (C23:0) | 74 | 1.8 | 7.8 | 9.5 | 28.7 |
| Methyl lignocerate (C24:0) | 382 | 0.6 | 8.6 | 13.9 | 42.4 |
| Methyl nervonate (C24:1n9) | 348 | 0.44 | 8.40 | 38.7 | 117.2 |

*Response factors (RF), expressed as mean value, were calculated for the methylated esters subsequently found in the plum seed oil samples. RSD means relative standard deviation; LOD means limit of detection; LOQ means limit of quantitation. The values of the response factor (RF) and relative standard deviation (RSD) were calculated as the average of 3 replicates. The limits of detection and quantification for each analyte corresponded to the concentration of the compounds that give a chromatographic signal (calculated as peak height), 3.3 and 10 times, respectively, higher than background noises. Bold letters are used for the corresponding data of those fatty acids present in the plum seed oil samples.

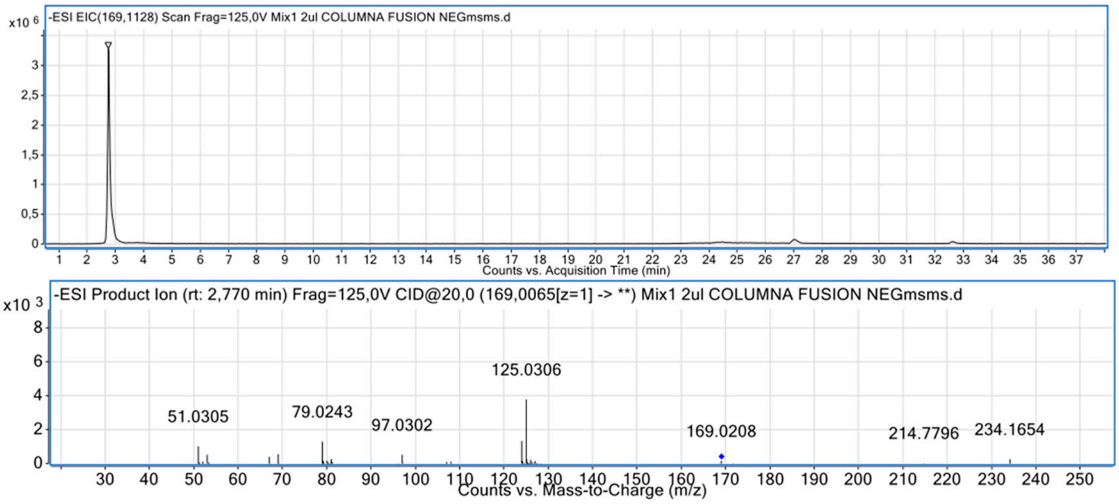


**Figure S7.** Gallic acid (standard solution 33 mg·L-1) chromatogram and mass spectrum obtained by high-resolution liquid chromatography coupled to a quadrupole time-of-flight mass spectrometer (HPLC-ESI-QTOF-MS) in SIM scan mode (Frag=125.0V). RT= 2.770 min.

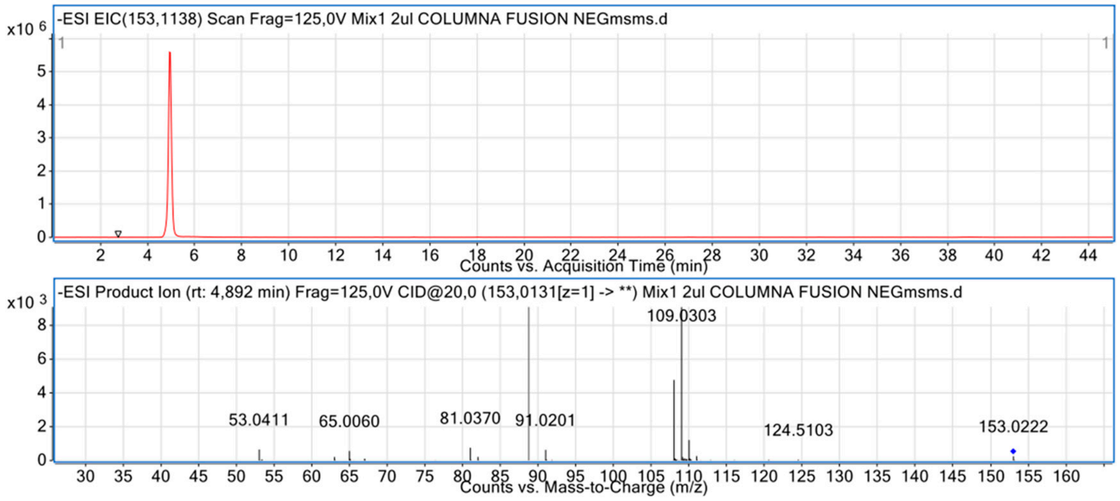


**Figure S8.** 2,3-Dihydroxibenzoic acid (standard solution 33 mg·L-1) chromatogram and mass spectrum obtained by high-resolution liquid chromatography coupled to a quadrupole time-of-flight mass spectrometer (HPLC-ESI-QTOF-MS) in SIM scan mode (Frag=125.0V). RT= 4.892 min.

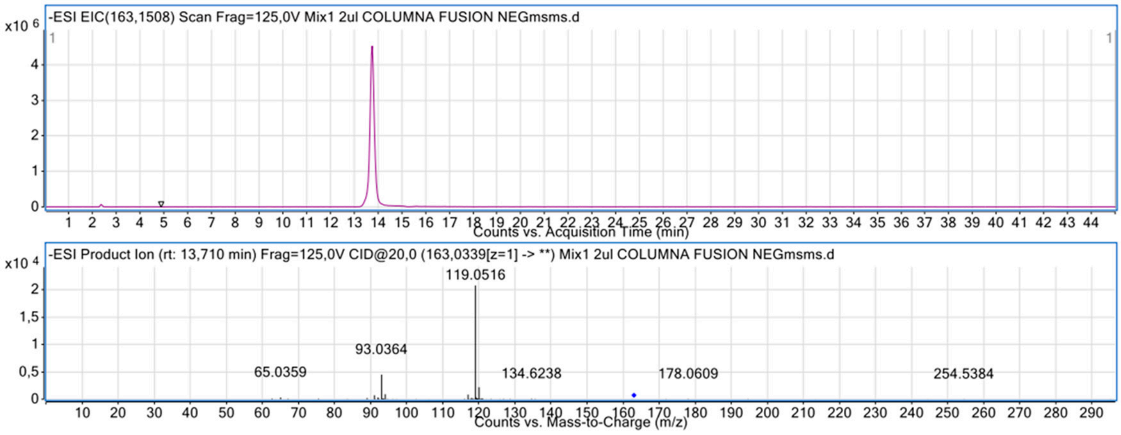

**Figure S9.** *p*-coumaric acid (standard solution 33 mg·L$^{-1}$) chromatogram and mass spectrum obtained by high-resolution liquid chromatography coupled to a quadrupole time-of-flight mass spectrometer (HPLC-ESI-QTOF-MS) in SIM scan mode (Frag=125.0V). RT= 13.710 min.

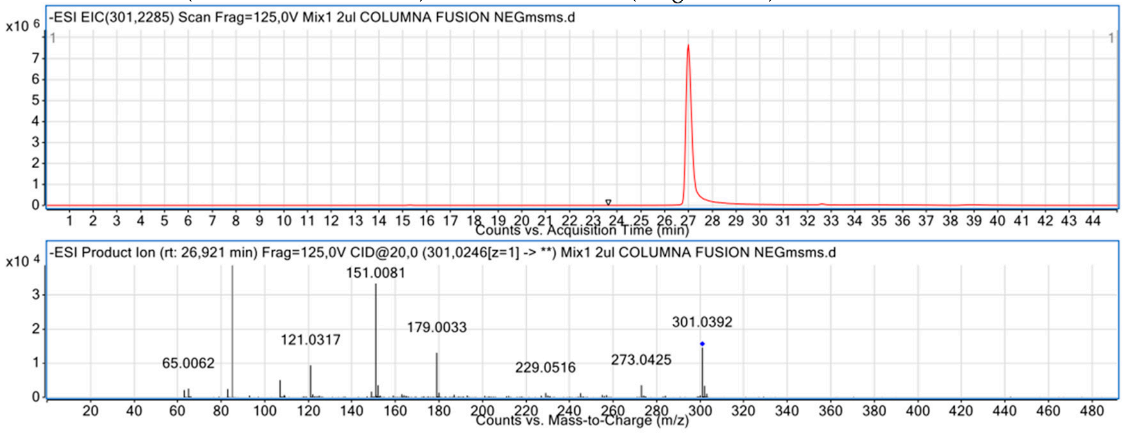


**Figure S10.** Quercetin (standard solution 33 mg·L$^{-1}$) chromatogram and mass spectrum obtained by high-resolution liquid chromatography coupled to a quadrupole time-of-flight mass spectrometer (HPLC-ESI-QTOF-MS) in SIM scan mode (Frag=125.0V). RT= 26.921 min.

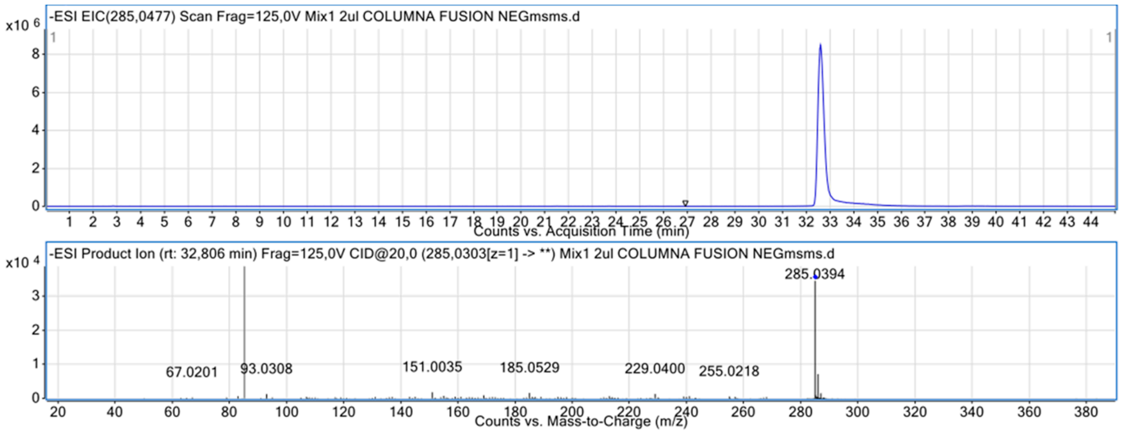


**Figure S11.** Kaempferol (standard solution 33 mg·L$^{-1}$) chromatogram and mass spectrum obtained by high-resolution liquid chromatography coupled to a quadrupole time-of-flight mass spectrometer (HPLC-ESI-QTOF-MS) in SIM scan mode (Frag=125.0V). RT= 32.806 min.

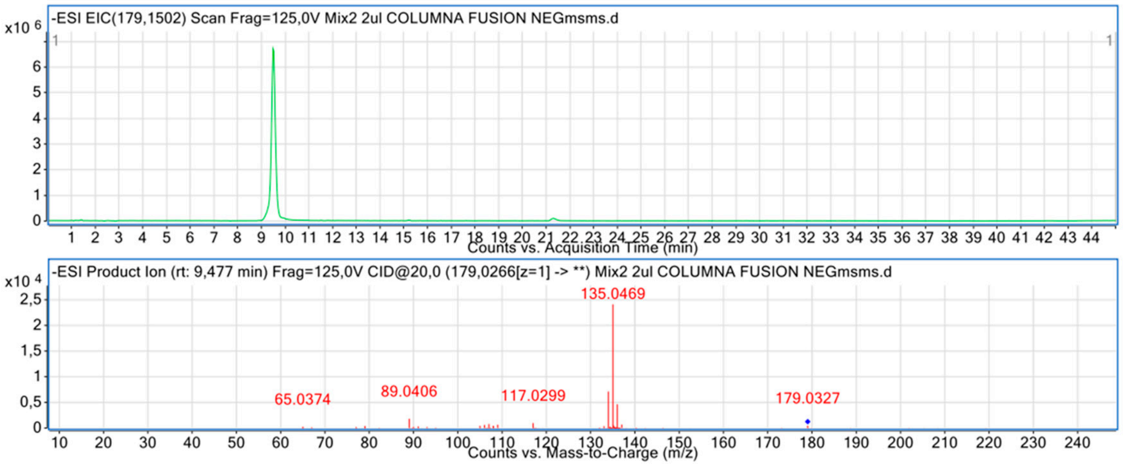


**Figure S12.** Caffeic acid (standard solution 33 mg·L$^{-1}$) chromatogram and mass spectrum obtained by high-resolution liquid chromatography coupled to a quadrupole time-of-flight mass spectrometer (HPLC-ESI-QTOF-MS) in SIM scan mode (Frag=125.0V). RT= 9.477 min.

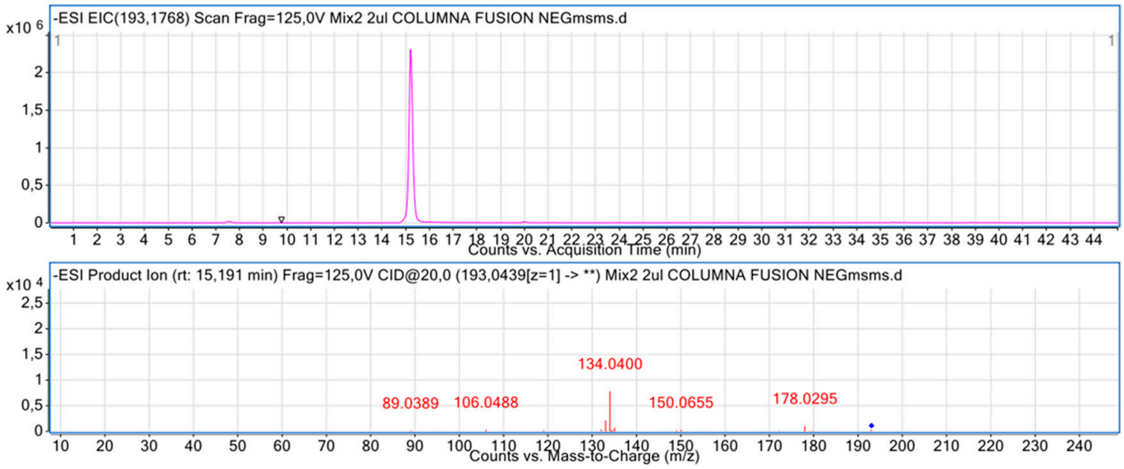


**Figure S13.** *trans*-ferulic acid (standard solution 33 mg·L-1) chromatogram and mass spectrum obtained by high-resolution liquid chromatography coupled to a quadrupole time-of-flight mass spectrometer (HPLC-ESI-QTOF-MS) in SIM scan mode (Frag=125.0V). RT= 15.191 min.

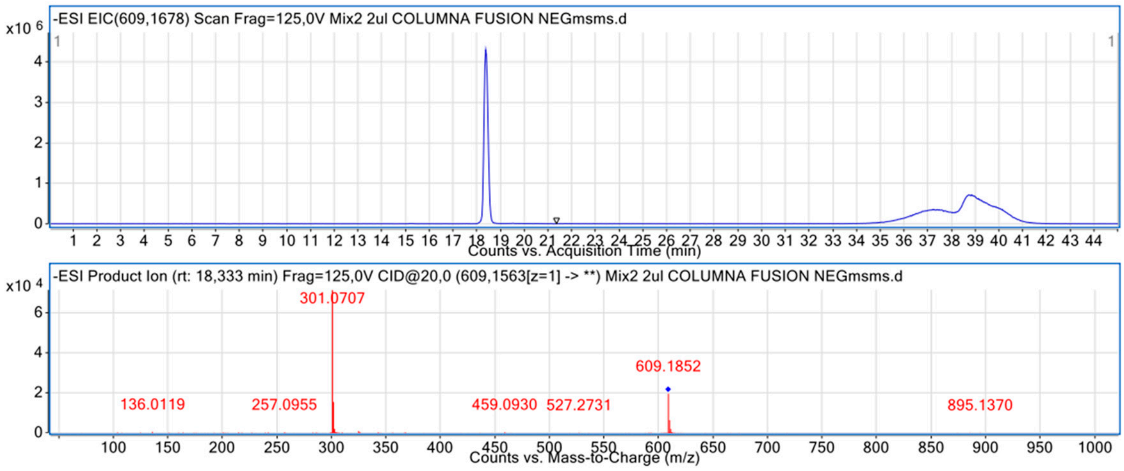


**Figure S14.** Hesperidin (standard solution 33 mg·L-1) chromatogram and mass spectrum obtained by high-resolution liquid chromatography coupled to a quadrupole time-of-flight mass spectrometer (HPLC-ESI-QTOF-MS) in SIM scan mode (Frag=125.0V). RT= 18.333 min.

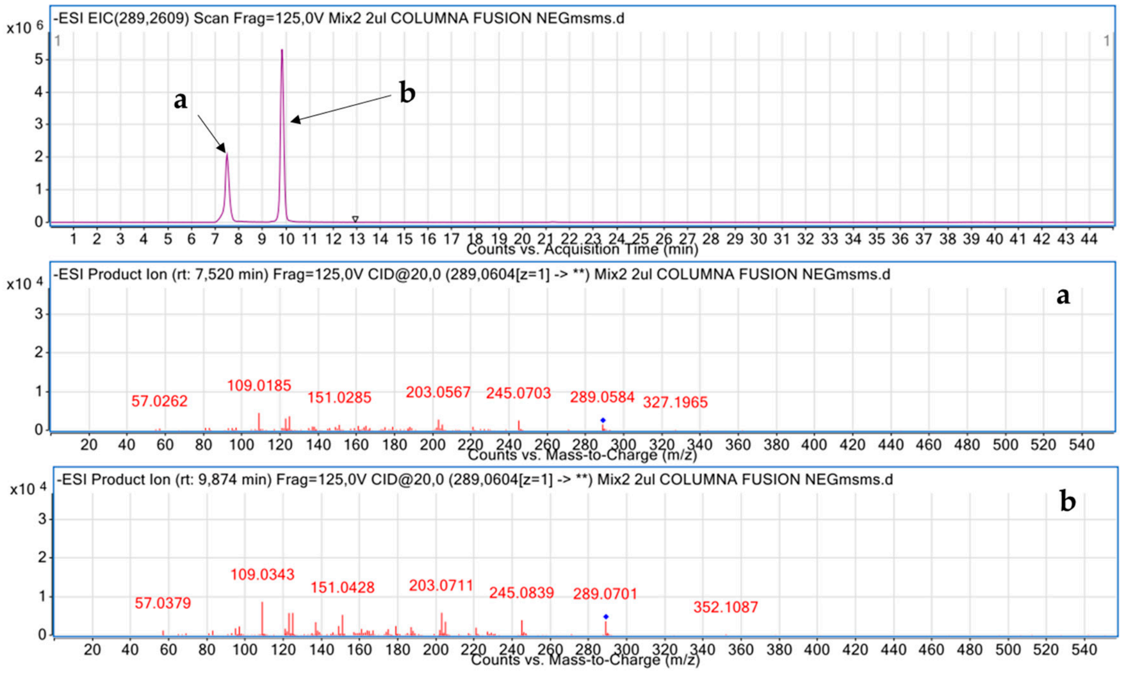


**Figure S15.** Catechin and epicatechin (standard solution 33 mg·L$^{-1}$) chromatogram and mass spectrums obtained by high-resolution liquid chromatography coupled to a quadrupole time-of-flight mass spectrometer (HPLC-ESI-QTOF-MS) in SIM scan mode (Frag= 125.0V). **a)** Catechin (RT= 7.502 min; m/z= 289.0584) and **b)** Epicatechin (RT= 9.874 min; m/z= 289.0701).

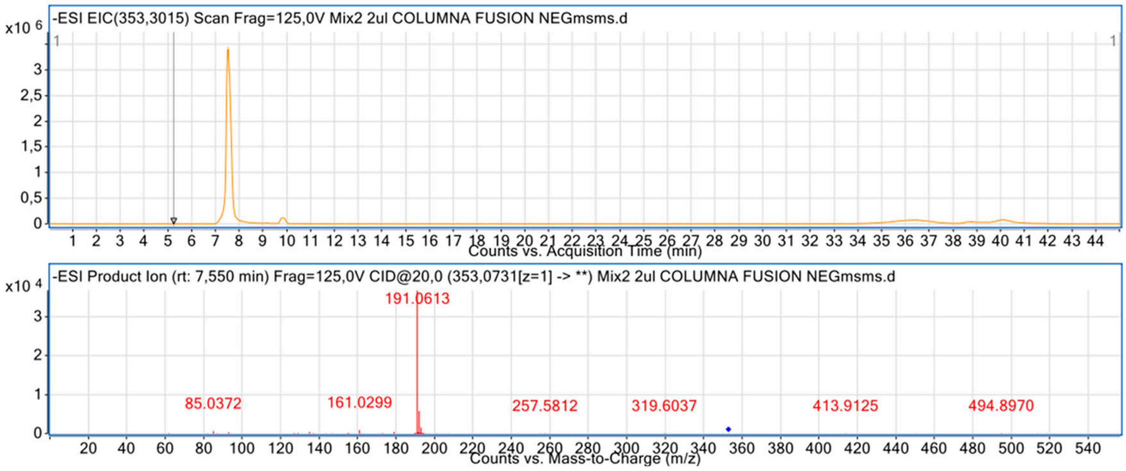


**Figure S16.** Chlorogenic acid (standard solution 33 mg·L$^{-1}$) chromatogram and mass spectrum obtained by high-resolution liquid chromatography coupled to a quadrupole time-of-flight mass spectrometer (HPLC-ESI-QTOF-MS) in SIM scan mode (Frag= 125.0V). RT= 7.550 min.

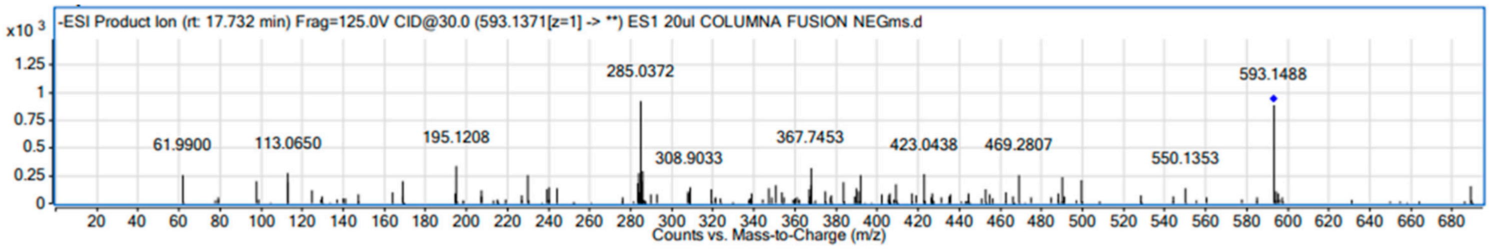


**Figure S17.** Kaempferol 3-rutinoside mass spectrum provided by FooDB database. RT= 17.232 min.

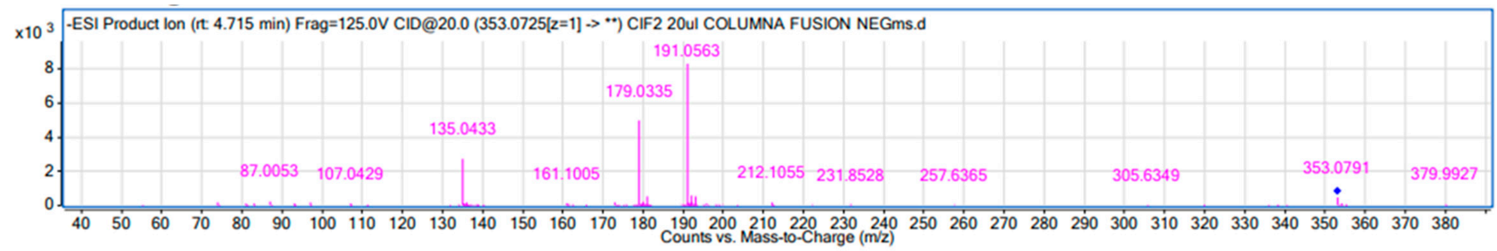


**Figure S18.** Neochlorogenic acid mass spectrum provided by FooDB database. RT= 4.715 min.

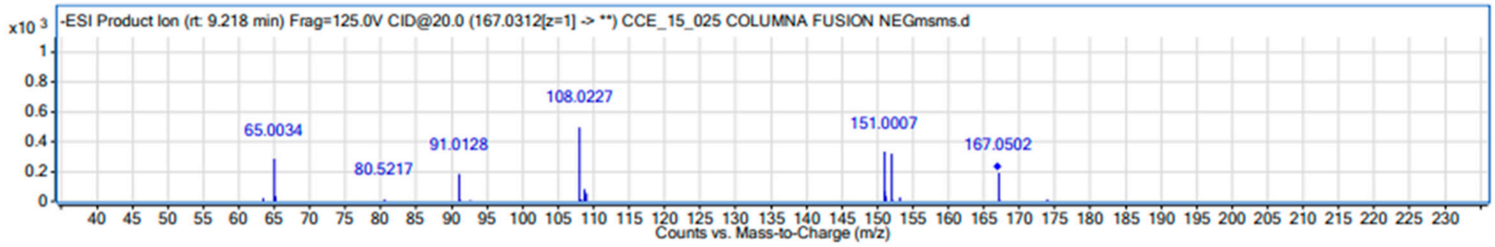


**Figure S19.** Vanillic acid mass spectrum standard solution. RT= 9.218 min.

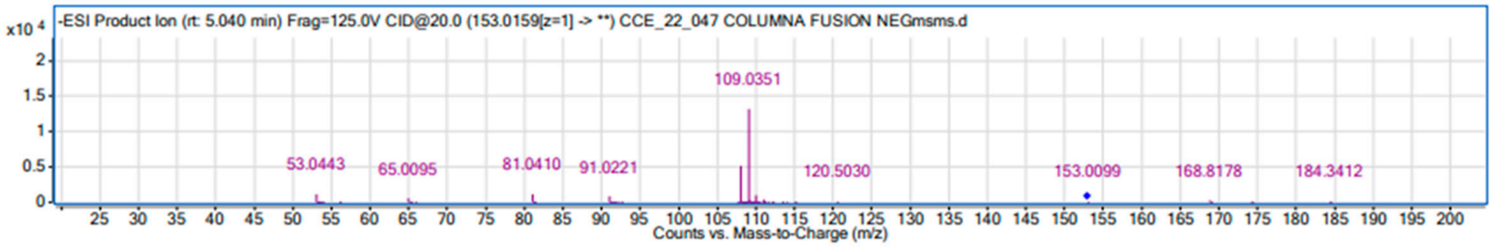


**Figure S20.** Protocatechuate mass spectrum provided by MassBank database. RT= 5.040 min.

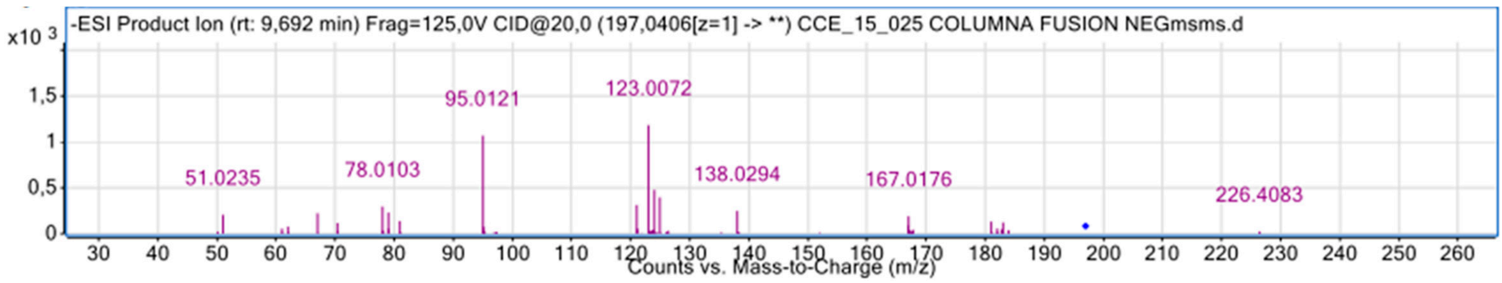


**Figure S21.** Syringic acid mass spectrum standard solution. RT= 9.692 min.

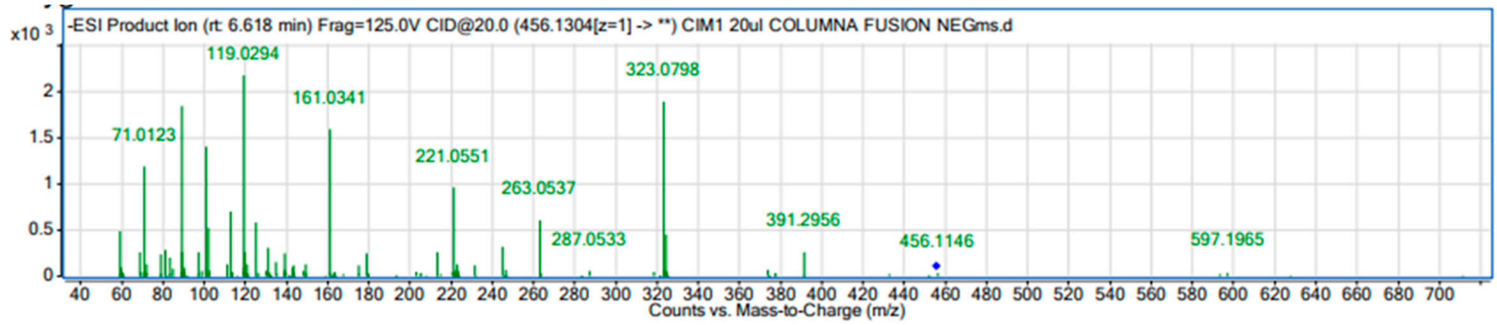


**Figure S22.** Amygdalin mass spectrum provided by FooDB database. RT= 6.618 min.

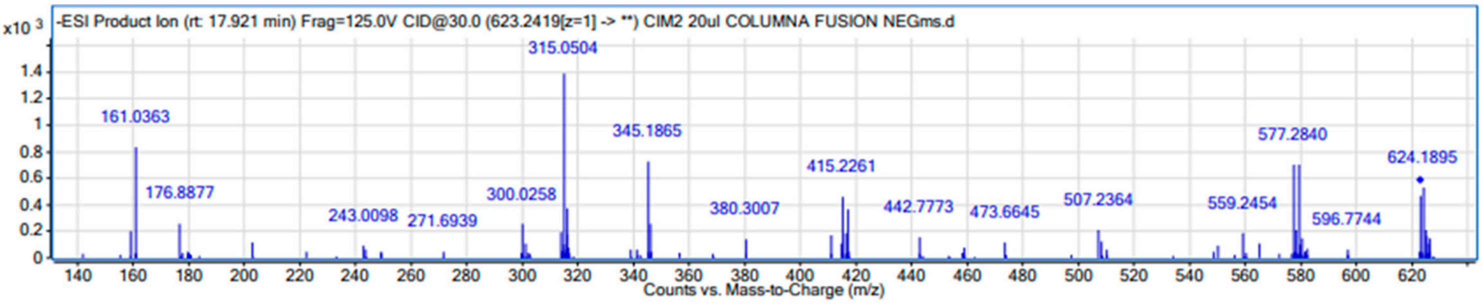


**Figure S23.** Isorhamnetin 3-rutinoside mass spectrum provided by MassBank database. RT= 17.921 min.

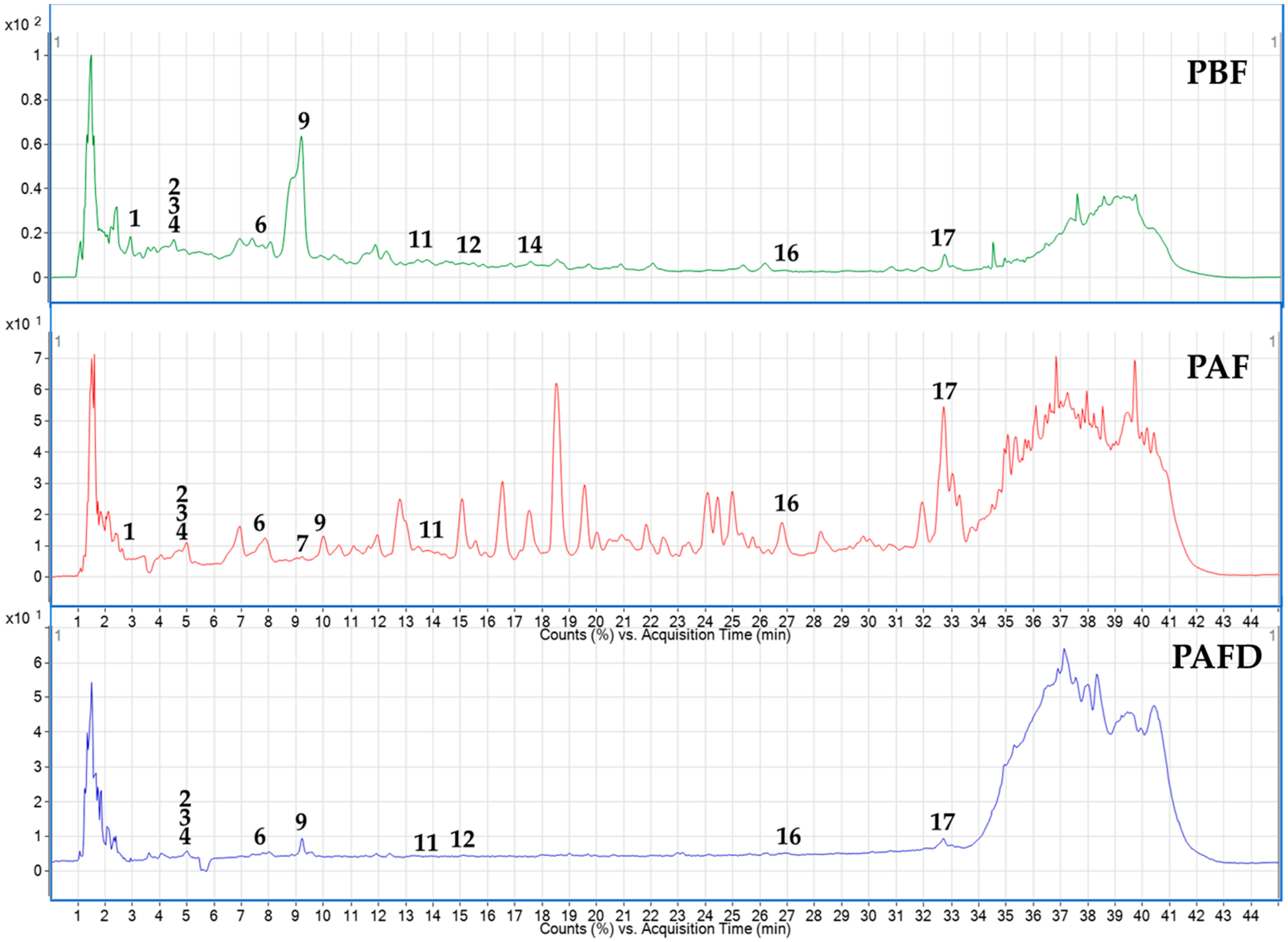


**Figure S24.** Chromatograms of polyphenols in defatted plum seed extracts by high-resolution liquid chromatography coupled to a quadrupole time-of-flight mass spectrometer (HPLC-ESI-QTOF-MS) in TIC negative scan mode (Frag= 125.0V). Peak identification: (**1**) gallic acid; (**2**) 2,3-dihydroxybenzoic acid; (**3**) neo-chlorogenic acid; (**4**) protocatechuate; (**6**) chlorogenic acid; (**7**) vanillic acid; (**9**) caffeic acid; (**11**) *p*-coumaric acid; (**12**) trans-ferulic acid; (**14**) isorhamnetin 3-rutinoside; (**16**) quercetin; (**17**) kaempferol.
